# Expertise in Coach Development: The Need for Clarity

**DOI:** 10.3390/bs13110924

**Published:** 2023-11-13

**Authors:** Christine Nash, Michael Ashford, Loel Collins

**Affiliations:** 1 Applied Coaching Science Research Group, University of Edinburgh, Edinburgh EH8 8AQ, UK; mike.ashford@ed.ac.uk (M.A.); loel.collins@ed.ac.uk (L.C.); 2 Grey Matters Performance Ltd., Stratford upon Avon CV37 9TQ, UK

**Keywords:** critical thinking, epistemology, knowledge, metacognition, reflection

## Abstract

In this position paper, we start by identifying the issues inherent to coach development; we then consider the current status of coach development and present our position before concluding with key points and suggesting resolutions for the issues. Our intention is to propose the progression of appropriate practices and approaches for the professional development and preparation of coaches. In coach development, a lack of clarity exists at both organisational and individual levels, particularly around the role of and aims for coach developers. Organisationally, we consider a radical reframing required to progress the profession of coach development. We also suggest that many individuals currently involved in coach development do not possess the requisite knowledge to move the field forward. Our aspirations for coach development include recognising the need for expertise and what it looks like in practice. Coaching and coach development interactions should examine particular coaching challenges, concentrating on the thought processes and decision-making strategies necessary to solve them. This necessitates a bespoke, problem-based approach to learning.

## 1. The Nature of Coaching

Coaching is complex and dynamic, often carried out in ill-structured and continually changing environments [1]. It is unsurprising that meeting the needs of developing coaches is equally challenging. The education of coaches has been extensively researched and reported over the last 20 years; however, the more all-encompassing role of the coach developer is a more recent extension to the coaching literature [1,2]. This addition to the coaching field requires greater investigation in order to provide clarity to those involved in coach development with individuals and organizations.

## 2. Organizational Views

The Chartered Institute for Management of Sport and Physical Activity (CIMSPA) defines coach developers as ‘expert support practitioners who plan for, implement, and sustain strategies and interventions in support of skilled performance in sport coaching’ [3] (p. 4). Challengingly, this definition, while providing some direction, seems at odds with others; for example, the European Coach Development Academy (ECDA) aims to “help to standardize the training of coaches and allow European values to be common and replicated in all EU countries” [4]. Similarly, the global organisation responsible for coaching, the International Council for Coaching Excellence (ICCE), considers that ‘Coach developers are not simply experienced coaches or transmitters of coaching knowledge. They are trained to develop, support and challenge coaches to go on honing and improving their knowledge and skills to provide positive and effective sport experiences for all participants’ [5] (p. 8).

These varied definitions, plus other national and multinational organisational classifications, fail to provide a clear picture of coach development and the role of coach developers. Importantly these definitions do not provide a meaningful consensus, nor do they provide the coherence required to move the field of coach development forward. Without conceptual clarity (cf., [6,7]), there is clearly potential for misunderstanding when considering the role and function of a coach developer, especially when designing and delivering coach development programmes, systems or strategies for coach learning. This clarity with respect to terms and purpose is key to moving the field of coaching and coach development forward in an effective and professional manner.

Much debate surrounds the current status of coaching, coach development and whether they are considered professions [8,9,10]. We are not suggesting that the practice of many coaches and coach developers is not professional, but more of an indictment against the lack of systematic support and the recognition and development of coaching expertise that would be aided by clarifying the role for the coach developer [11]. Professionalism, in this case, is an attitude or approach which we believe is essential for the wider field of coaching to adopt in order to progress. This approach has been adopted over a period of time within the medical field [12,13]. We accept that coach development operates at multiple levels of sophistication across and within countries and organisations; however, there are numerous examples of inconsistency.

## 3. The Need for Consistency

UK Sport is a proponent of bespoke coach development within the high-performance area [14]; nevertheless, this message does not appear to be actioned across sports within the four countries of the UK. However, the Nordic countries present a more coherent picture, with Norway and Finland providing examples of ‘joined-up thinking’. Coach development is identified as a priority in Finland, recognised as an essential element of growing their sport system, and subsequently receives attention and funding [15]. Similarly, Norway has a very well-defined and regulated coach development process, leading to the success of their Olympiatoppen and Trenerløftet, high-performance-coach development and talented-coach-development programmes, respectively [16]. Further afield, the Australian Government supports coach development through the auspices of the Australian Sport Commission [17] at the community coach level as well as in high-performance. These instances exemplify the differences in approaches and degrees of support for coach development offered globally [18,19,20].

Therefore, it is important to clarify the differences between education, development and training and instruction. All are important to the ongoing learning of a coach; however, their application is dependent on the context of that coach’s development—different settings requiring different approaches. Like effective coaching, effective coach development is a matter of ‘it depends’, but the key is in what it depends on [2]. For clarity, coach education describes formal, classroom-based experiences in which participants follow a prescribed set of learning activities over a period of time. It is commonly offered by national sporting organisations or coaching bodies and considered, cynically, by some to be a money-making endeavour [21]. Equally, instruction can be likened to a set of specific guidelines on how to complete a task, enabling the completion of the task by following the directions but not requiring an understanding of why the task needs to be completed or which approach is used. More broadly, coach development is an all-encompassing term that includes education, instruction as well as other developmental approaches, allowing for an individualised, nuanced approach agreed upon between the coach and coach developer. Coach development, therefore, includes formal, non-formal and informal learning opportunities which are again determined by the needs of the coach and the context of their development. Coach development is a longer-term process underpinned by a relationship between a coach and a coach developer and is designed to change as practice evolves. Key attributes associated with coach development such as expertise, judgment, challenge, coaching, mentoring, scaffolding, reflection, exploration and articulation differentiate coach development from other approaches. Attempts have been made to parameterise the role of a coach developer (cf., [22]), offering a mechanistic, process-driven capture of the coach development process. Whilst we appreciate the intent behind this process, we would argue that this mechanistic approach can be too limiting to truly meet coaches where they are.

## 4. Aspirations to Expertise

There does appear to be a confusion of terms, with education, training and development being used interchangeably in many instances. We propose, for the avoidance of doubt, that coach development should be focused on the development of expertise, accepting that there are constraints to adopting this approach so, at present, it may be aspirational. This expertise approach requires an understanding of the conception of both knowledge and learning, as highlighted by Collins, Abraham and Collins [11], but we are still not able to define an expert coach developer with clarity and authority. The key element here is that coach developers are able to recognize the different points that coaches are at in their learning journey—a comprehension of the situation in which the development is taking place. Put simply, meeting the coach where they are. For example, consider the approaches needed to work with coaches who want black and white ‘facts’ (similar to the instruction mentioned earlier) as opposed to coaches who are able to grasp ‘what if’ scenarios and explore the various options that actual practice presents. Within education, Entwistle and Peterson [23] refer to dualistic, multiplistic and relativistic conceptions of knowledge as existing on a continuum. Dualism, as espoused by Perry [24], is considered to be the most basic form of assumption that someone may propose and is often compared to viewing knowledge as right or wrong. We suggest that coaches and coach developers would not be able to function effectively within the complex dynamic of coaching practice with this perspective; however we have had experience of that type of approach. Multiplism is the next stage of development and applies to those who may question or feel uncertain about decisions that they make. Multiplicity by its definition suggests there are many different choices and authority figures, such as coach educators and mentors within organisations, who are not the only ones with answers [25]. The final category, relativism, is what we want coaches to reach when they have learned to think critically despite challenges [26]. Here, coaches will have learned to analyse different sources, reason, logic and evidence, recognising that practices and perceptions should be questioned when they are faulty.

Kahneman and Klein [27] consider that with the appropriate expertise approach, individuals become more skilled at recognising, accepting and dealing with uncertainties and, as a result, are more likely to deepen their thinking and progress their practice. Coach developers, therefore, need an understanding of how to facilitate deep thinking in themselves and their coaches, challenging assumptions, encouraging problem solving and widening their knowledge base, using evidence informed materials rather than a ‘gut feeling’ or *just* opinion. However, we would suggest that the ‘gut feeling’ improves over time if appropriate levels of deep thinking have taken place (cf., [28]).

A well-informed coach development workforce would be of significant benefit to organisations wishing to develop highly skilled, motivated coaches capable of continual learning who are ‘agile’ in response to situations and provide the best experience possible. In order to develop expert coaches, coach developers themselves must aspire to be expert coach developers, displaying the following characteristics:

⮚ Expertise is domain-specific and developed over time. Therefore, coach developers must have extensive and carefully crafted experiences in specific areas, such as learning strategies, that reflect their deep understanding of the area.
⮚ Experts are able to structure knowledge for easy access. Coach developers must ‘sift and sort’ their vast knowledge into an organisational structure that enables retrieval with little attentional effort.
⮚ Experts develop routines for simple tasks to free up working memory. Coach developers need to ‘chunk’ information into small units to enable greater use of short-term memory.
⮚ Experts recognise patterns faster than novices. Coach developers must to recognise key features of emerging situations based on previous experience and solutions, mostly using schemas.
⮚ Experts take deeper meaning from cues. Expert coach developers need to notice key features and meaningful patterns of information that can be missed or not recognised as important by non-experts.
⮚ Experts sort problems into categories based on the features of their solution. Experts categorize problems based on deep meaning and an understanding of the area and tend to work backwards from a solution perspective [28].

**Figure 1:**
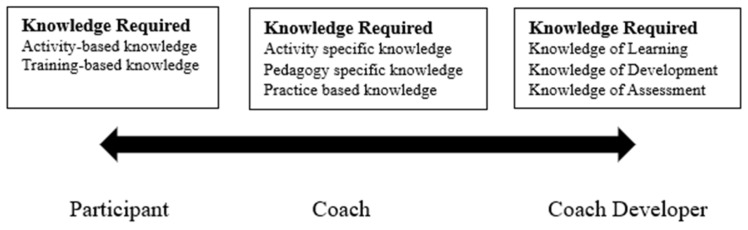
Typical coach developer pathway.

We argue that aspiring to expertise is different to *just* being a good coach developer. Currently, these characteristics do not appear to be recognised in the recruitment and deployment of coach developers, as presented in Figure 1. Many coach developers follow a simple continuum, moving from player to coach to coach developer in a hierarchical, linear progression.

While this pathway may work for many, especially in heavily structured systems, there is little recognition of the different knowledge and skills required for each—they are related but different at the same time. The false assumptions of a good athlete becoming a good coach have long been acknowledged, although it does still occur [29,30]. Using the same logic, we suggest that being a good coach does not equate to being a good coach developer. Whether by accident or design, the systems in place for coach development do not facilitate the transition from one to another. At the heart of the challenge is the incongruity between coaching development, coaching practice and the organisational constraints imposed by sporting organisations that muddies the waters.

We also believe that there is an urgent need for an expanded range of developmental tools, especially if we wish to meet the needs of the coaches where they are at—there is no one-size-fits all. At the heart of this argument are two fundamental questions—what is good coaching, and what is good coach development? However, before these questions can be answered, we need to establish clarity. The clarity of what constitutes the role of a coach developer needs to link to similar clarity in what coach developers aim to achieve.

## 5. Epistemological Confusion

For the coach developer, achieving role clarity is inherently difficult. Frequently, the coach developer sits on a metaphorical see-saw between the strategic focus of a sport or system and their wider socio-political goals, balanced with the operational focus of an individual coach’s day-to-day practice [11,31]. This incorporates wider stakeholders, including individuals in positions of leadership, partners of the sporting system, such as funding services or agencies, and the wider interdisciplinary team that supports coaches and athletes, all of which contribute to how a coaching ‘system’ may operate [32]. Thus, the coach developer is required to operate at two levels. There is the *top-down* level, which captures the big-picture focus of a system, the system’s philosophy, goals and direction and the organisation’s epistemological position [33]. In contrast, the *bottom up which* captures the perceived values, beliefs, habits and actions of each individual they interact with, a personal-epistemological system, or the individual coach’s position. Unfortunately, simplified conceptions of naïve coaching ‘philosophies’ are littered amongst coach development experiences, which causes a lack of coherence between these *top-down* and *bottom-up* interactions [34]. Instead, more of an emphasis should be placed in the idea of ensuring coherence between the epistemological positions of a coach, a coach developer and an organisation.

Perry [35] suggested that awareness of epistemology is fundamental to learning as it supports individuals in understanding how we think, perceive, value and learn about knowledge [23]. Interestingly, by way of some practical examples, research has rarely explored the coherence or integration of an epistemology which flows from a top-down collective strategy for improving coaching knowledge. For instance, the strategy established by the Rugby Football Union is, from the bottom up, made up of a collection of individual rugby coaches and the coach development workforce available to them. Furthermore, limited thought is given to the consideration of an appropriate level of desirable variability between individual beliefs and values at different stages of a system, nor their coherence with a top down epistemological position within coach learning. If we consider how these ideas apply to a real-world context such as a Talent Development Pathway (TDP), they may have a guiding philosophy which might include (i) the maximization of the potential of those within their system, (ii) a progression of at least three athletes per year into the senior world-class system and (iii) the delivery of a positive athlete experience for all involved regardless of the outcome. However, this TDP also forms part of a wider governing body and agency that prescribes goals, medal expectations and progression expectations per year. Therefore, it is not surprising that the coach developer, when supporting coaches in meeting these expectations whilst also balancing the coaches’ individual learning needs, knowledge development and practice, can experience a melting pot of *epistemological confusion*. In essence, coach developers are often faced with a conundrum of either meeting organisational demands or a coach’s development needs.

Epistemological confusion relates closely to Argyris and Schön’s [36] idea of *espoused theories*, which are the values, beliefs, proposed intentions, actions and behaviours we believe we uphold in practice vs. our *theories in use*—those actually displayed in practice. Recently, research was conducted to explore these ideas from a top-down view (cf., [37]) and a bottom-up view [38] within both practical and applied settings. Hall et al. [37] investigated the alignment between academy football coaches’ individual beliefs and the academy system’s overarching ‘philosophy’, which they felt guided how they supported players’ development throughout the system. Through systematic observations and interviews, these authors highlighted that a substantial gap existed between the academies’ espoused philosophy and the interactions in use within coaching sessions. Hall et al. [37] also suggested that ‘philosophy’ was used as a buzzword by participants throughout the investigation, with limited adherence to how coaches should behave. Similar findings were unearthed by Ashford and colleagues [38] at an individual coach level with the theories espoused by academy rugby union coaches and what they intended to do, extracted from semi-structured interview data, versus the theories in use, demonstrated through systematic observations of training sessions and self-confrontation interviews. This study found that coaches were often guided by ideological conceptions of coaching practice, driven through top down expectation, which rarely played out in the realities of their coaching. However, these findings from team sports differ from those of Mees et al. [39], who demonstrated that the attraction for outdoor instructors working within organisations was the alignment of philosophy and practice. Therefore, these studies suggest that whether the perspective is bottom-up or top-down, it is the environment that plays a key role.

Close parallels can be made between Argyris and Schön’s work [36] and that of Collins and colleagues in exploring the epistemological chain (EC) [40,41]). Collins et al. [41] (p. 227) described the EC as a “consistent, logical relationship between philosophy, modus operandi, aims and session content at macro, meso and micro levels”. If we extend the idea of an EC to the top-down epistemological position that many sports and systems adopt within coach learning and development, it means that we are no longer referring to a chain but complex, interconnected epistemological pathways that converge on one another at moments in time. For example, when a coach developer first interacts with a group of Olympic athletic coaches who work across different disciplines with different athletes. Unfortunately, research would suggest that coherent, integrated epistemological positions, supported through coach development, education and instruction, are seldom identified in the sporting world [42]. In fact, the state of play would suggest quite the opposite. By way of example, sports which hang their hat on a single approach, “game based” or “implicit vs. explicit” approaches towards coaching and coach development, are likely to result in *epistemological confusion* throughout this system (cf., [43,44]) or a perceived lack of value for a particular approach [39]. We strongly suggest that these two top-down positions represent the naïve conception of a philosophy expressed by Hall et al. [37], and it is unlikely that coaches are upholding these epistemological beliefs at the coalface, practicing from a sophisticated epistemological position. Unfortunately, therefore, such positions will likely result in regular and sizable *epistemological voids*, such as an unsophisticated and naïve assumption by a coach developer, or coach, that anything other than game-based practice is obsolete as a coaching method [43].

Rather than hanging our hats on a particular approach to coach development, it seems logical to explore and investigate the coherence, integration and understanding of epistemology as a coherent framework that supports practice. In particular, what do systems want their coach development to contain and why, and how will that be coherent with those it is aimed at supporting? We feel it is important to add a caveat here that a wider, evidence-informed diet of content, under the umbrella of a pragmatic view that ‘everything works somewhere, nothing works everywhere’ [45] (p. 63), is also a sound place to start. For example, a coach developer may seek to support a boxing coach in zooming in on clips taken from video and audio footage of their coaching. This video could present a tendency to intervene too much and offer ‘white noise’ to the athlete, much of which is unable to be used. Whilst this method of building self-awareness for a coach may be useful in this context, it may be confronting and overly disruptive for others; therefore, more subtle methods may be more appropriate for meeting coaches where they are.

To endeavour to understand the epistemological position, voids and degree of coherence throughout a system, a deeper understanding of the political, economic, social, technological, legal and environmental [46] factors may serve to exemplify the true levels of coherence and integration between coach development, coaching and the practitioner workforce [47]. It is important to consider how this applies practically to real-world settings, for instance, a sporting organisation may choose to conduct a research project auditing the coherence and integration of the workforce’s epistemological position from top-down and bottom-up standpoints. They may investigate the following: (1) the socio-political goals set by leadership and their trickle-down effect on the actions and behaviours of the workforce, with coach developers as a central mechanism; (2) a consideration of the amount of funding allocated to coach development, how that funding is distributed between formal, non-formal and informal learning experiences and their perceived impact; (3) an exploration of social interactions within learning experiences and the coherence and integration between them within different stages of the sport and/or between individual coach developers; (4) how technology, resources and materials are used to support coach development and their impact; (5) from a legal standpoint, the ethical practices of those within, across and between the workforce; and (6) how together, these five aforementioned factors amalgamate to form an environment, and the possible instances of epistemological coherence, integration, confusion and voids and their wider impact on behaviour.

## 6. The Challenges for the Coach Developer

The challenge for the coach developer is in facilitating the development of adaptable coaches—those that can be agile in response to novel demands that are routinely encountered. Indeed, the only aspect of coaching that *is* routine is the uniqueness of the interaction with an athlete. As alluded to earlier, by their nature, good coaches are adaptable, skilled and agile thinkers [48,49]. With this in mind, investigations of coach thinking and decision making have shown that decision making and critical reasoning guide practice and have much to do with how learning experiences unfold for the coach [43,50].

Consequently, coach development can be challenging. There is no single way to teach a coaching technique and no single way that a coach may learn that technique. Multiple development decisions need to be navigated; therefore, coach development is complex and undoubtedly non-linear. Logically, then, coach development cannot, and indeed should not, be scripted or proffer a prescribed set of routines or a single solution or approach. However, coach development still must retain coherence with epistemological political, economic, social, technological, legal and environmental demands. This is dependent on the coach developer making a series of nested decisions about their educational practices and making choices about a particular course of action to facilitate development within the demands of those constraints (cf., [51]). Furthermore, the coach developer must also develop the requisite knowledge domains in order to even recognise key cues and decision-making instances in the first place, linking to our characteristics of expertise presented earlier [31,52]. For example, if we consider a coach developer within an elite team sport context, for them to first recognize and second provide feedback on the limited speed and representativeness of skill attempts within a small-sided game, they must possess the prior knowledge and understanding of what game speed actually looks, sounds and feels like!

From this perspective, coach development is dynamic, demanding and problematic—it must be responsive to the situational demands of a given context and the coach within it. The effective coach developer embraces this complexity and requires constant innovation, flexibility and adaptability—the agility mentioned earlier. Logically, then, the endpoint of a coach’s developmental process is an adaptable and creatively thinking coach.

However, as we have stated, coach development cannot be just a top-down process; even at the initial levels of coach development, the experiences of trainee coaches are a crucial aspect of a given coach’s development. Indeed, we argue that neophyte coaches may actually benefit from ‘trying’ to coach in a safe, supervised and supported context before they are supported to develop. This experience, even as an ‘assistant’ provides a context for their learning. Such approaches have inherent complexities; single visions of coaching and learning only serve to mask inherent complexities and make simplistic what should only be made as simple as possible but no simpler. Oversimplification is an unfortunately common pitfall for the novice coach developer and plagues low-level coaching awards designed by the NGBs, though it is unclear if this is by design or accident. Certainly, there comes a point in simplified systems that a coach must make the leap from procedure, or process, to adaptability. Such a leap highlights the epistemological void cited earlier but can hinder coach development. Consider two practical and regularly applied scenarios in practice. First, a coach developer is shown how to deliver a particular coach education programme; the training takes the same duration as the actual course, and the reality is that they are shown what and how to deliver the programme rather than why. Or consider another situation in which a coach developer is able to deliver coach education to the level they are qualified at, passing on just what they know from their own training and how it may be used based on their own experience by replicating what they have been shown. In either scenario, a prescriptive syllabus supports the delivery of an identical programme that may be little more than indoctrination into the ‘systems way’. Quality is simply measured using the degree to which the coach developer matches the model presented and their ability to replicate or mimic rather than apply and adapt, illustrating the desired and sought-after adaptability. This would seem little more than the naïve epistemological stance also discussed earlier.

## 7. Creating the Adaptable and Creatively Thinking Coach

Hatano and Inagaki [53] contrast adaptive and routine expertise, noting that both, for the coach and coach developer, demand the capacity to perform standard tasks and functions without error. They differentiate routine expertise in two ways: firstly, as competence with a task’s parts or functional units, and secondly, as an ability to manage low variability in that particular task. Like routine, adaptive expertise shares domain-specific and metacognitive skills but differs in a need for innovation driven by context [53,54,55]. Adaptive expertise is further characterised by efficiency and innovation in applying knowledge when approaching novel situations and challenges [53,56,57], as mentioned earlier. The flexible, creative and innovative use of the competencies found in routine expertise enables adaptability [58].

More recently, Ward et al. [59], Mees et al. [60] and Pulakos et al. [61] all suggested that adaptability may be the essential ingredient in expertise per se, without which expertise could not happen—‘Conditio Sine Qua Non’ [59] (p. 35)—suggesting that the routine-adaptive dichotomy is unwarranted. Mees et al. [60] also suggested a spectrum of adaptability based on the dynamic nature of the performance environment. However, this may also be a simplistic solution as adaptability seems non-linear and more dendritic or network-like, an increasing bandwidth of adaptability in which the more adaptiveness gained, the more can be considered in an exponential manner. Practically, Hutton et al. [57] suggest that adaptability has several aspects: (1) high degrees of situational comprehension, (2) possessing a range of skills to draw on as options in that situation and (3) self-awareness to balance situational demands with individual abilities. These aspects, in turn, require a comprehension of the interaction between those components and an epistemology that acknowledges and values adaptability and new knowledge. Consequently, adaptive performance is multi-dimensional and relevant to particular roles and contexts; therefore, we suggest that coaching and coach development are two independent entities. Logically, environments that require adaptation and flexibility should, therefore, require adaptive experts or experts with greater adaptability. Conversely, those environments or tasks that do not require adaptation and flexibility do not require adaptive experts, thus linking the epistemological position cited earlier. Naïve epistemological stances, chains and pathways have the potential to work in non-dynamic environments; equally, dynamic environments probably require sophisticated epistemological stances, chains and pathways [62].

Consequently, coach development may be best-suited to a range of approaches that facilitate situated development in which the *What* of a curriculum is also supported by a comprehension of *How* and *Why* coaching skills are developed and enhanced in particular ways. It is for this reason that good coaches may not make good coach developers, although, as will become clear, it can be an advantage.

## 8. Adaptability and Creativity Requires Good-Quality Thinking

Explicit to adaptability is the coaches’ ability to select and appraise a range of knowledge sources, make sense of that knowledge when combined with their experiences, and, importantly, consider that evidence and how it may inform their coaching in the present and future [26]. Selecting and appraising relevant knowledge and its potential application can be a daunting task—of course, this can be easy if the coach developer themselves is working from a script! However, scripted coach development develops script-driven coaches.

This position, if rationally considered, is not surprising given that we know coaches perceive that they learn most about coaching by engaging in it; they seem attuned to the ‘how’ and why more than the ‘what’ [63,64,65]. Consequently, the notion of apprenticeship may be better suited to coach development. Apprenticeship has been influential in teaching and learning throughout history, especially in trades and professions [66]. Apprenticeship has value in that it is highly situated, frequently in the workplace, under the tutelage of a master and takes place over an extended period of time. Therefore, the situated learning and facilitation of skills regularly associated with apprenticeships would fit nicely with the support of coaches in responding to the cognitive challenges they regularly face in practice [67]. Such cognitive challenges associated with coaching processes, are aspects not typically ‘visible’ that can be developed alongside the practical. Apprenticeships frequently employ four elements in the pursuit of expertise development [68]: (1) *content*, the types of knowledge required for expertise (domain knowledge and metacognitive strategies); (2) *methods*, ways in which the development of expertise can be facilitated (modelling, coaching, scaffolding, articulation, reflection, and exploration, see Table 1); (3) *sequencing*, the ordering of activities to promote development; and (4) *sociology*, the social characteristics of the learning environment.

The coach can be engaged in the dynamic region beyond their current ability, their Zone of Proximal Development (the Zone of Proximal Development refers to the difference between what an individual can achieve by themselves with no help versus their achievement with the help of a teacher or their peers) [71], with support from a coach developer, a ‘master’ coach, developing cognition and practice in context [72]. However, this development is reliant on the ‘master’ recognising and understanding their own coaching and being able to articulate why, drawing on the evidence, practice and experience and, importantly, their metacognition [73].

Modelling, coaching and scaffolding are tools that would be familiar to a practicing coach and are typical in any traditional apprenticeship. However, the others, articulation, reflection and exploration, are used with the intention of developing mental models, macrocognitive and metacognitive skills, rather than a focus on physical skills [68]. These latter three characterise the developmental approach as a cognitive apprenticeship. Principally, the cognitive skills being learned are the underpinning skills which are not entirely observable and may be tacit in nature but are key to enabling the development of adaptability and creativity. The intention here is to make these cognitions visible to the learner via the coach developer articulating their thoughts, role and function—the why. For example, supporting a fencing coach in understanding what good blade work feels like at the point of contact is an extremely difficult skill to develop in a coach; therefore, a careful orchestration of methods to support their understanding is essential. One positive element of scaffolding for cognitive apprenticeships is the functioning within a community of practice in which a shared language and set of mental models representing good coaching and good coach development would exist [66].

A practical way to manage this challenge is to focus coach development on the growth of critical thinking skills in response to day-to-day cognitive challenges [74]. Critical thinking skills enable the coach to focus on the most appropriate sources of information, the highest-quality evidence and knowledge to inform their practice. Logically, then, critical thinking is a goal for coach development using this method. However, there is a need to foster a coaching culture that includes critical thinking and supports coaches in being both analytical and critical. This necessitates a willingness to ‘throw stones at false idols!’ [26] (p. 129) while also accepting that many of the assumptions made about coach development may not hold—criticality should be effectively applied to the practices of coach and coach developer alike [75]. A well-informed and professional coach development workforce [76] must be professional in its critique of the knowledge and knowledge sources that it uses to inform its practices [26,50,77].

## 9. Creating Coach Developers as Critical Consumers of Coaching Knowledge

A critically thinking coach developer must position information and ideas within the larger picture of coaching and the organisation’s epistemology, as highlighted earlier. By being critical of the content they have presented or have been asked to present as part of a syllabus, they can comprehend the strengths and weaknesses of a particular approach and identify evidence that supports or contradicts its potential application.

Importantly, the critically thinking coach developer can problem solve and adapt to changing circumstances, such as coach needs, and is therefore as pedagogically agile as the coach they develop. This leads coach developers and coaches to focus their attention on particular coaching episodes and challenges they may face on a day-to-day basis and to critically consider initial actions, situational assessment, cues and potential errors in response to them (cf., [67,78]). Coaches are already critical, for example, recognising the effectiveness of an intervention based on observed performance is a critical evaluation [79]. Also consider the coach selecting from various approaches based on recommendations from another coach when this advice is, at least, considered worthy of trial and then critical appraisal before critiquing its effectiveness. Linked to the creativity highlighted earlier is an essential evaluation of any information’s appropriateness based on its intended use and how it has been derived, interpreted and presented. Thus, an essential part of the adaptable, critically thinking coach developer is their capacity to learn from their experiences; in other words, reflection.

## 10. The ‘R-Word’

Nash and colleagues [79] report that many coaches do not perceive themselves as reflective practitioners, a point Collins and Eastabrook [26] agree with, highlighting that coaches are more involved in the ‘in-action’ thinking, addressing the problem at hand. This is in contrast to the ‘on-action’ reflection [80] that academic models often offer [81] or those typically endorsed in coach-development programmes. Coaches and coach developers have frequently given reflection ‘a go’ and found it not to have value, so they discount it. However, they recognise the need to be adaptable and flexible, which requires them to integrate critical thinking within the coaching process; therefore, managing this cognitive dissonance is a key challenge for the coach developer as it is desirable in the developmental process.

However, a pedagogy of coach development is more than defining how to act. It is more about creating conditions through which coaches learn about adaptability, creativity and thinking about their coaching in a goal-directed manner. As a consequence, the contents of any developmental curriculum need to be framed to ensure they are understood and in such a way that they can be effectively combined. Frequently, if the contents are limited but interchangeable and can also be interlinked, it has an impact on curriculum design as well as substance and delivery. Effective coach development facilitates the growth of effective reflective skills as well as resourcefulness, a resilience and capacity to work independently or within a team often known as reciprocity or learning to learn (see Learnacy [82]). It seems that coaching developers and development programmes need to create opportunities for mentoring coaches and modelling coaching practice, while also scaffolding those opportunities. Extending coach development opportunities that enable the coach to reflect on and explore their practice while also having the ability to articulate what, how and why an approach is selected and applied. Importantly, creating an environment in which adaptability and criticality are valued and encouraged and ultimately enabling a space in which adaptability can be explored and articulated by the coach and coach developer are key to achieving expertise. Thus, to consider the application of these ideas in a practical setting, coach development curriculums which begin with theoretical content and rely on a coach’s effective translation of theory into practice may steer coaches away from this desired end. Instead, more problem- and challenge-focussed curriculums, bespoke to specific coach episodes faced by coaches on a day-to-day basis whilst still being coherent and integrated within the agreed epistemological approach, are more likely to support coaches in progressing towards a level of expertise through effective reflection [83].

## 11. Thematic Discussion

This paper was fuelled by a desire to help advance appropriate practices and approaches to the professional development and preparation of coaches. It is designed as a presentation of our thoughts to encourage debate and hopefully move the field of coach development forward. We realise we have presented some complex information which is not always supportive of coach development in its current format(s). We also are mindful that there are many coaches and coach developers who would share our view that there is a need to change—we need expert coach developers advancing the number of expert coaches. We use this section to address some of the key questions arising from the evidence that we presented.

### 11.1. Why Do We Need/Not Currently Have Clarity?

We are strongly advocating the need for clarity—clarity of the role of a coach developer is key to effective performance. As we have shown, there are a variety of definitions for the role of coach developer which often evolve to fit the needs of an organisation or sport rather than the needs of coaches. We suggest that as coach development is a new and evolving field for many organisations, clarity may not be apparent in the system, as the system may not have progressed to meet this new area. It would appear that in many cases, the role of the coach developer has been ‘shoehorned’ to fit existing systems and available resources, contributing to the lack of clarity.

We do not have a universally accepted definition of a coach developer that clearly articulates roles and aim. The definitions from organisations we presented earlier demonstrate a wide range of interpretations, leaving uncertainty as to the scope of the role. This further complicates the recruitment and deployment of coach developers as well as how they are prepared to fulfil their remit of developing sport coaches to work effectively within the dynamic environments in which they operate. Much of this can lead to the epistemological confusion that we referenced earlier.

Key Takeaways:

- An accepted definition of the coach developer;
- Coherent articulation of the role and aims of the coach developer.

### 11.2. How Will Clarity Help?

We stated that our purpose here is to help the field of coach development move forward. Clarity is instrumental in defining goals and creating steps to achieving them while making effective decisions. Without this, there may be a lack of motivation, indecisiveness and a lack of direction or focus—none of which will help coach developers, coaches or sporting organisations. We need to ensure that the underlying philosophy, or mission statement, of an organisation is clearly communicated to everyone in the organisation. The values, beliefs, proposed intentions, actions and behaviours that define an organisation must also be visible in a coach developer’s practice and be coherent with their activities and functioning. By having this clarity of purpose, it becomes easier to concentrate on some outcomes—expert coach developers producing expert coaches.

Key Takeaways:

- Clarity provides conceptual and practical understanding;
- Clarity enables focus on the key outcome.

### 11.3. Why Are We Not Drawing on Research in Other Fields?

We have looked extensively at what works in other areas, such as education, medicine, and military contexts, in which people operate in dynamic environments. We have especially concentrated on evaluating the development of expertise within different domains. The shifting of perspective from dualism to relativism [23] demonstrates the benefits of considering coach development from a variety of viewpoints, asking questions, solving problems and being able to synthesise all the available options.

The apprenticeship model, especially cognitive apprenticeship, has been shown to add value within the field of education, but this has also expanded to include businesses and the medical environment. Coaches value coach developers working with them in the coaching/practice environment, so this model would appear to tick the box of situated learning. The social environment is crucial to the success of apprenticeship, enabling access to examples of good practice and varying levels of expertise opportunities to model behaviour, seek advice and understand that multiple solutions are possible. This environment highlights the importance of the clarity and coherence of purpose from all involved in the process. Before we wholeheartedly embrace and adopt these strategies, and many others, we need to carefully evaluate whether these approaches would work in coach development—remember the earlier words ‘everything works somewhere, nothing works everywhere’ [45] (p. 64).

Key Takeaways:

- Research in other fields is well-established and evidence-informed;
- We need to be more open to new ideas and approaches.

### 11.4. Why Are We Not Promoting the Benefits of Reflection and Critical Thinking?

We highlighted the importance of both reflection and critical thinking in the development of expertise—again, we can draw on work in other domains as to the success of these strategies. Why is sport coaching different? Reflection is mandatory within teaching, and the ability to think critically is an integral element of study within higher education. Reflection can ensure that everyone is benefitting from the learning experience, learning at their own pace and ensuring motivation to continue learning. Reflective practice can improve problem-solving skills and address challenges, allowing coach developers to devise individual strategies to meet the needs of each learner. The implication of focussing on particular coaching episodes and cognitive challenges is one regularly suggested across the expertise literature (cf., [78,84]) and offers coaches more value with respect to the reflective process. In this way, reflection can encourage innovations in how to improve a coach’s practice in reference to specific coaching problems, demonstrating that reflection and innovation fully complement each other. Interestingly, reflecting in this way supports individuals in engaging in skills which align closely to the characteristics of experts mentioned previously in this paper (cf., [28]). Thus, critical thinking enhances reflection and vice versa as it allows for the gathering of knowledge, information processing and the analysis of data. According to Sternberg and Halpern [85] (p. 132), ‘critical thinking allows people to solve problems more creatively, independently, and effectively’. Given the reported benefits of reflection and critical thinking, why would we not incorporate both as key elements of coach development?

Key Takeaways:

- Reflection and critical thinking are essential to developing expertise;
- Reflection and critical thinking require buy-in and practice to be effective.

### 11.5. Is Simplicity Helping Coaches?

From both research findings and anecdotal reports, there does appear to be a mandate from coaching organisations to break information down into simple parts that can be easily presented and digested. We suggest that this is doing a disservice to the majority of coaches who want to develop themselves, their athletes and their teams. Is this simplicity for the benefit of the coaches or coach developers? We frequently highlighted the complexity of coaching and the challenges faced by coaches and coach developers in trying to embrace the adaptability required to consider all the possibilities and select the most appropriate approach to a situation. This is not a simple task.

The skills required of a coach developer, contained in Table 1, are complex and require considerable investments of time and energy to practice. We also suggest that coach developers should accumulate the characteristics of expertise necessary to operate at this level. We expect coach developers to observe coaches in the workplace, engage with them around their practice and conceive strategies to help them improve within their own context. This cannot be broken down into a series of simple undertakings. As put forward earlier, we stand by the statement that coach development services and coach developers should aim to make information and learning as *simple as it can be, but no simpler!*

Key Takeaways:

- Coaching and coach development are complex constructs;
- Breaking elements down into simple components does not reflect the complexity of the coaching role.

### 11.6. What Is an Expert Coach Developer?

This is a very difficult concept to define—a bit like trying to nail jelly to a wall! We mentioned a ‘master’—an individual who is able to identify problems and who can make effective judgements by using all their knowledge, techniques and experiences to facilitate, enrich, and deepen learning to improve coaching practice. We emphasised the need for adaptive expertise for coach developers due to the complexities of practice and the need for the flexibility to shift approaches based on the needs of the coach in front of them. This series of questions were designed to highlight some of the key features required for expertise, underlining the challenges of achieving this goal.

If coaches are required to possess a depth and breadth of knowledge in order to respond to key challenges (cf., [28,51]), the coach developer’s knowledge must exceed that of the coach to support critical thinking and reflection as to where improvements can be made. Consequently, an understanding of these knowledge forms, specific to their coaching context, is essential. Research in this area, therefore, must attempt to investigate and elicit coaching and coach developer expertise through the appropriate integration of different methods. Interestingly, Nash and Collins [28] made the recommendation that a Cognitive Task Analysis (a Cognitive Task Analysis refers to a research method that uncovers and makes sense of what people know and how they think) methods and derivatives of it (e.g., an applied cognitive task analysis) would be a sufficient way of meeting this end. However, since this recommendation, only three studies of note have been conducted in a coaching context (cf., [67,86,87]). This leads us to suggest to a *call to action* to identify the minimum requirements for experience, learning, breadth and depth of knowledge across different domains which coach developers should possess in order to fulfil their role professionally and to ensure that professional standards are maintained in these positions.

Key Takeaways:

- Expertise is hard to attain but often conferred on those who are very good rather than expert;
- We need coach developers who can encourage adaptive expertise in coaches.

## 12. In Summary

This is an overview of our views, beliefs and our position on how coaches should be supported by coach developers. Having posed and hopefully answered these questions arising from our thoughts, we would suggest that coach development requires a radical rethink. Our position advocates clarity, starting with a shared language and common ideas from which to bridge the sizeable gap between the current state of coach development and our aspirational vision. We require thoughtful coach developers, striving to achieve expertise to produce thinking coaches capable of adapting to the vagaries of practice.

We hope to offer an evidence-informed position from which to make the following suggestions:

- We need to re-align our coach development systems to ensure clarity at all levels;
- Coach development systems need to be built on pedagogical, macrocognitive and metacognitive skills;
- We need to develop a coherent framework that supports practice rather than being ideologically wedded to one approach;
- We need coach developers to meet the needs of the coaches in the place they are at;
- We aspire to expertise—this is an approach that needs to be adopted by all;
- Research in this area should support a deepened understanding of coach and coach developer expertise to support all of the above.

## Figures and Tables

**Table 1:** Cognitive apprenticeship methods (adapted from Collins [68]).

Developmental Approach	Description
**Modelling**	A demonstration of the skills, usually by an expert, to enable the learner to develop a mental model of the skill. Often includes verbalising the cognitive processes.
**Coaching**	Observing the learner and offering challenges, support and feedback
**Scaffolding**	Putting supports in place to enable the learner to carry out the task in their ZPD. Scaffolding must be highly personalised, and if done poorly can have a negative effect on the learner emotionally [69].
**Articulation**	The learner separates, verbalises and demonstrates their understanding of the component knowledge, reasoning and thinking processes in a domain
**Reflection**	Learners compare their own thought processes and problem solving with that of others, in particular, that of an expert
**Exploration**	Giving learners time and space to problem solve on their own, akin to a problem-based learning approach [70], fading supports and self-setting of goals

## Data Availability

Data are contained within the article.

## References

[1] McQuade S., Nash C. (2015). The Role of the Coach Developer in Supporting and Guiding Coach Learning. Int. Sport Coach. J..

[2] Collins D., Taylor J., Ashford M., Collins L. (2022). It depends coaching—The most fundamental, simple and complex principle or a mere copout?. Sports Coach. Rev..

[3] Chartered Institute for Management of Sport and Physical Activity (CIMSPA) (2021). CIMSPA Professional Standard: Coach Developer.

[4] European Coach Developer Academy. https://e-cda.eu/.

[5] International Council for Coaching Excellence (2016). International Coach Developer Framework, Version 1.1.

[6] Beedie C., Benedetti F., Barbiani D., Camerone E., Cohen E., Coleman D., Davis A., Elsworth-Edelsten C., Flowers E., Foad A. (2018). Consensus statement on placebo effects in sports and exercise: The need for conceptual clarity, methodological rigour, and the elucidation of neurobiological mechanisms. Eur. J. Sport Sci..

[7] Eys M.A., Schinke R.J., Jeffery S.M. (2007). Role perceptions in sport groups. Group Dynamics in Exercise and Sport Psychology.

[8] Nash C. (2022). The coaching professional. Practical Sports Coaching.

[9] Powell S.M., Fasczewski K.S., Stevens N., Tocci N.D., Jewell S., Blumberg J., Cangas M.A. (2022). Pressure, Stress, and Coping: Ex-ploring the Professional Demands of NCAA Division I Coaching. J. Sport Behav..

[10] Duffy P., Hartley H., Bales J., Crespo M., Dick F., Vardhan D., Nordmann L., Curado J. (2011). Sport coaching as a ‘profession’: Challenges and future directions. Int. J. Coach. Sci..

[11] Collins D., Abraham A., Collins R. (2012). On Vampires and Wolves—Exposing and exploring reasons for the differential impact of coach education. Int. J. Sport Psychol..

[12] Jha V., Bekker H.L., Duffy S.R., Roberts T.E. (2007). A systematic review of studies assessing and facilitating attitudes towards professionalism in medicine. Med. Educ..

[13] Ng S.L., Forsey J., Boyd V.A., Friesen F., Langlois S., Ladonna K., Mylopoulos M., Steenhof N. (2022). Combining adaptive expertise and (critically) reflective practice to support the development of knowledge, skill, and society. Adv. Health Sci. Educ..

[14] UK Sport (n.d.) Coach Development. https://www.uksport.gov.uk/learning-and-development/coach-development.

[15] Hämäläinen K., Blomqvist M. (2016). A New Era in Sport Organizations and Coach Development in Finland. Int. Sport Coach. J..

[16] (2019). Olympiatoppen Olympiatoppens Modellfor Utvikling av Trenerei Norsk Toppidrett.

[17] Australian Government/Australian Sports Commission, Coaching. https://www.ausport.gov.au/coaching.

[18] Nash C. (2023). Career Development of Elite Coach Developers. Developing Sports Coaches.

[19] Mills J.P., Denison J. (2018). How power moves: A Foucauldian analysis of (in)effective coaching. Int. Rev. Sociol. Sport.

[20] Cope E., Cushion C.J., Harvey S., Partington M. (2021). Investigating the impact of a Freirean informed coach education programme. Phys. Educ. Sport Pedagog..

[21] Nash C., Sproule J. (2012). Coaches’ perceptions of their coach education experiences. Int. J. Sport Psych..

[22] Muir B., North J. (2023). Supporting Coaches to Learn Through and From Their Everyday Experiences: A 1: 1 Coach Development Workflow for Performance Sport. Int. Sport. Coach. J..

[23] Entwistle N.J., Peterson E.R. (2004). Conceptions of learning and knowledge in higher education: Relationships with study behaviour and influences of learning environments. Int. J. Educ. Res..

[24] Perry W.G. (1970). Forms of Ethical and Intellectual Development in the College Years: A Scheme.

[25] Myers S.A. (2010). Using the Perry Scheme to Explore College Student Classroom Participation. Commun. Res. Rep..

[26] Collins L., Eastabrook C., Nash C. (2023). The importance of critical thinking in coaching: Separating the wheat from the chaff. Developing Sports Coaches.

[27] Kahneman D., Klein G. (2009). Conditions for intuitive expertise: A failure to disagree. Am. Psychol..

[28] Nash C., Collins D. (2006). Tacit Knowledge in Expert Coaching: Science or Art?. Quest.

[29] Taylor J., Collins D. (2020). The Highs and the Lows—Exploring the Nature of Optimally Impactful Development Experiences on the Talent Pathway. Sport Psychol..

[30] Nash C.S., Sproule J. (2009). Career Development of Expert Coaches. Int. J. Sports Sci. Coach..

[31] Abraham A., Collins D., Morgan G., Muir B., Lorenzo A., Ibanez S.J., Ortega E. (2009). Developing Expert Coaches Requires Expert Coach Development: Replacing Serendipity with Orchestration. Aportaciones Teoricas Y Practicas Para El Baloncesto Del Futuro.

[32] Burns A., Collins D. (2023). Interdisciplinary practice in performance sport: A scoping review of evidence of collaboration. Eur. J. Sport Sci..

[33] Abraham A., Allison W., Abraham A., Cale A. (2016). Task Analysis of Coach Developers: Applications to The FA Youth Coach Educator Role. Advances in Coach Education and Development From Research to Practice.

[34] Schommer M. (1994). Synthesizing epistemological belief research: Tentative understandings and provocative confusions. Educ. Psychol. Rev..

[35] Perry W.G., Chickering A.W. (1981). Cognitive and ethical growth: The making of meaning. The Modern American College: Responding to the New Realities of Diverse Students and a Changing Society.

[36] Argyris C., Schon D. (1974). Theory in Practice: Increasing Professional Effectiveness.

[37] Hall J., Cope E., Townsend R.C., Nicholls A.R. (2022). Investigating the alignment between coaches’ ideological beliefs and academy philosophy in professional youth football. Sport Educ. Soc..

[38] Ashford M., Cope E., Abraham A., Poolton J. (2022). Coaching player decision making in rugby union: Exploring coaches espoused theories and theories in use as an indicator of effective coaching practice. Phys. Educ. Sport Pedagog..

[39] Mees A., Collins L. (2022). Doing the right thing, in the right place, with the right people, at the right time; a study of the development of judgment and decision making in mid-career outdoor instructors. J. Adventure Educ. Outdoor Learn..

[40] Collins L., Collins D., Grecic D. (2014). The epistemological chain in high-level adventure sports coaches. J. Adventure Educ. Outdoor Learn..

[41] Collins L., Collins D. (2015). Integration of professional judgement and decision-making in high-level adventure sports coaching practice. J. Sports Sci..

[42] Crowther M., Collins D., Collins L., Grecic D., Carson H.J. (2022). Investigating academy coaches’ epistemological beliefs in red and white ball cricket. Sports Coach. Rev..

[43] Nash C., Taylor J. (2021). ‘Just Let Them Play’: Complex Dynamics in Youth Sport, Why It Isn’t So Simple. Front. Psychol..

[44] Smith K., Burns C., O’neill C., Duggan J.D., Winkelman N., Wilkie M., Coughlan E.K. (2022). How to coach: A review of theoretical approaches for the development of a novel coach education framework. Int. J. Sports Sci. Coach..

[45] Wiliam D. (2016). Leadership for Teacher Learning Creating a Culture Where All Teachers Improve so that All Students Succeed.

[46] Martinez-Contreras R.M., Hernandez-Mora N.C., Vargas-Leguizamon Y.R., Borja-Barrera S.M. (2022). PESTEL Analysis and the Porter’s Five Forces: An Integrated Model of Strategic Sectors. Handbook of Research on Organizational Sustainability in Turbulent Economies.

[47] Pereira F., Salvi M., Verloo H. (2017). Beliefs, Knowledge, Implementation, and Integration of Evidence-Based Practice Among Primary Health Care Providers: Protocol for a Scoping Review. JMIR Res. Protoc..

[48] Elliott J.M., McCullick B.A. (2019). Exceptional adaptability in collegiate coaching. Sports Coach. Rev..

[49] Mees A., Sinfield D., Collins D., Collins L. (2020). Adaptive expertise—A characteristic of expertise in outdoor instructors?. Phys. Educ. Sport Pedagog..

[50] Stoszkowski J., MacNamara Á., Collins D., Hodgkinson A. (2020). “Opinion and Fact, Perspective and Truth”: Seeking Truthfulness and Integrity in Coaching and Coach Education. Int. Sport Coach. J..

[51] Abraham A., Collins D. (2011). Taking the Next Step: Ways Forward for Coaching Science. Quest.

[52] Kirschner P.A., Hendrick C. (2020). How Learning Happens: Seminal Works in Educational Psychology and What They Mean in Practice.

[53] Hatano G., Inagaki K., Stevenson H., Azuma H., Hakuta K. (1986). Two courses of expertise. Child Development and Education in Japan.

[54] Crawford V.M., Schlager M., Toyama Y., Riel M., Vahey P. Characterizing adaptive expertise in science teaching intro-duction and overview. Proceedings of the American Educational Research Association Annual Conference.

[55] Hatano G., Oura Y. (2003). Commentary: Reconceptualizing School Learning Using Insight From Expertise Research. Educ. Res..

[56] Bransford J., Derry S., Berliner D., Hammerness K., Beckett K.L., Darling-Hammond L., Bransford J. (2005). Theories of learning and their roles in teaching. Preparing Teachers for a Changing World: What Teachers should Learn and be Able to Do.

[57] Hutton R., Ward P., Gore J., Turner P., Hoffman R., Leggatt A., Conway G. Developing adaptive expertise: A synthesis of literature and implications for training. Proceedings of the 13th International Conference on Naturalistic Decision Making.

[58] Trotter M.J., Salmon P.M., Goode N., Lenné M.G. (2017). Distributed improvisation: A systems perspective of improvisation ‘epics’ by led outdoor activity leaders. Ergonomics.

[59] Ward P., Gore J., Hutton R., Conway G.E., Hoffman R.R. (2018). Adaptive skill as the Conditio sine qua non of expertise. J. Appl. Res. Mem. Cogn..

[60] Mees A., Toering T., Collins L. (2021). Exploring the development of judgement and decision making in ‘competent’outdoor in-structors. J. Adv. Educ. Outdoor Learn..

[61] Pulakos E.D., Arad S., Donovan M.A., Plamondon K.E. (2000). Adaptability in the workplace: Development of a taxonomy of adaptive performance. J. Appl. Psychol..

[62] Elby A., Hammer D. (2000). On the substance of a sophisticated epistemology. Sci. Educ..

[63] Nelson L.J., Cushion C.J. (2006). Reflection in Coach Education: The Case of the National Governing Body Coaching Certificate. Sport Psychol..

[64] Sinfield D., Allen J., Collins L. (2019). A comparative analysis of the coaching skills required by coaches operating in different non-competitive paddlesport settings. J. Adventure Educ. Outdoor Learn..

[65] Stoszkowski J., Collins D. (2014). Communities of practice, social learning and networks: Exploiting the social side of coach development. Sport Educ. Soc..

[66] Lave J., Wenger E. (1991). Situated Learning: Legitimate Peripheral Participation.

[67] Taylor J., Ashford M., Jefferson M. (2023). High performance coach cognition in the wild: Using applied cognitive task analysis for practical insights–cognitive challenges and curriculum knowledge. Front. Psychol..

[68] Collins A., Sawyer R. (2005). Cognitive Apprenticeship. The Cambridge Handbook of the Learning Sciences.

[69] Bean T.W., Stevens L.P. (2002). Scaffolding Reflection for Preservice and Inservice Teachers. Reflective Pr..

[70] Lonergan R., Cumming T.M., O’Neill S.C. (2022). Exploring the efficacy of problem-based learning in diverse secondary school classrooms: Characteristics and goals of problem-based learning. Int. J. Educ. Res..

[71] Vygotsky L.S. (1978). Mind and Society: The Development of Higher Mental Processes.

[72] Dennen V.P., Jonassen D.H. (2004). Cognitive Apprenticeship in Educational Practice: Research on Scaffolding, Modeling, Mentoring, and Coaching as Instructional Strategies. Handbook of Research on Educational Communications and Technology.

[73] Barry M., Collins L. (2021). Learning the trade—Recognising the needs of aspiring adventure sports professionals. J. Adventure Educ. Outdoor Learn..

[74] Al-Kumaim N.H., Alhazmi A.K., Mohammed F., Gazem N.A., Shabbir M.S., Fazea Y. (2021). Exploring the Impact of the COVID-19 Pandemic on University Students’ Learning Life: An Integrated Conceptual Motivational Model for Sustainable and Healthy Online Learning. Sustainability.

[75] Cushion C. (2022). Changing Police Personal Safety Training Using Scenario-Based-Training: A Critical Analysis of the ‘Dilemmas of Practice’ Impacting Change. Front. Educ..

[76] Culver D.M., Werthner P., Trudel P. (2019). Coach Developers as ‘Facilitators of Learning’ in a Large-Scale Coach Education Programme: One Actor in a Complex System. Int. Sport Coach. J..

[77] Tiller N.B. (2022). From debunking to prebunking: How skeptical activism must evolve to meet the growing anti-science threat. Skept. Inq..

[78] Klein G., Calderwood R., MacGregor D. (1989). Critical decision method for eliciting knowledge. IEEE Trans. Syst. Man Cybern..

[79] Nash C., MacPherson A.C., Collins D. (2022). Reflections on Reflection: Clarifying and Promoting Use in Experienced Coaches. Front. Psychol..

[80] Schön D.A. (1983). The Reflective Practitioner: How Professionals Think in Action.

[81] Collins L., Collins D. (2018). The role of ‘pracademics’ in education and development of adventure sport professionals. J. Adventure Educ. Outdoor Learn..

[82] Claxton E. (2002). Building Learning Power: Helping Young People Become Better Learners.

[83] Nash C., Martindale R., Collins D., Martindale A. (2012). Parameterising expertise in coaching: Past, present and future. J. Sports Sci..

[84] Hoffman R.R. (2007). Expertise Out of Context. Proceedings of the Sixth International Conference on Naturalistic Decision Making.

[85] Sternberg R.J., Halpern D.F., Lilienfeld S.O., Basterfield C., Bowes S.M., Costello T.H., Baron J., Bensley D.A., Bernstein D.A., Braasch J.L.G. (2020). Critical Thinking in Psychology.

[86] Downes P.W., Collins D. (2021). Exploring the Decision Making Processes of Early Career Strength and Conditioning Coaches. Int. J. Phys. Educ. Fit. Sports.

[87] Downes P., Collins D. (2021). Examining the Roles and Consequent Decision-Making Processes of High-Level Strength and Conditioning Coaches. Societies.

